# Supplementary material for: The Identification of Metabolites and Effects of Albendazole in Alfalfa (Medicago sativa)
Source: Int J Mol Sci. 2020 Aug 18;21(16):5943. doi: 10.3390/ijms21165943 (PMC7460629; doi:10.3390/ijms21165943)
Supplement: Supplementary file 1 [file ijms-21-05943-s001.pdf]

A, FULL MASS SPECTRUM OF *m/z* 444.14 CE -25 eV with fragmentation ion *m/z* 208, 240, 282, and 250.

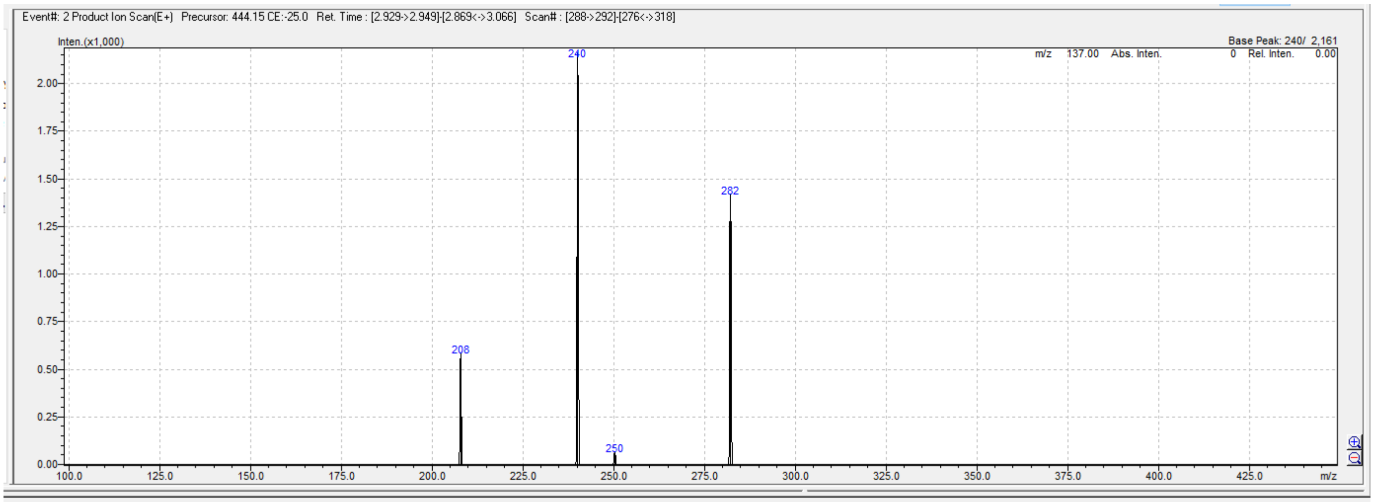

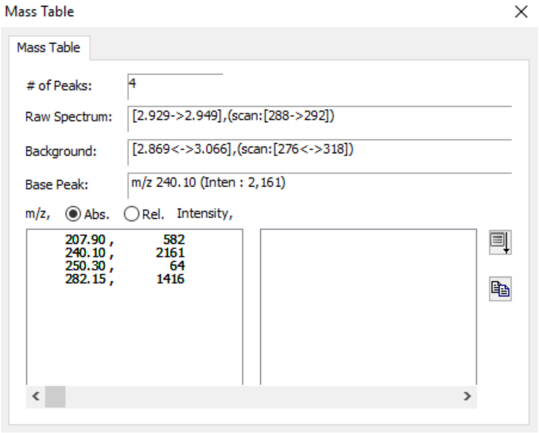

B, FULL MASS SPECTRUM OF 428.14 CE -25 eV with fragmentation ion *m/z* 234, and 266

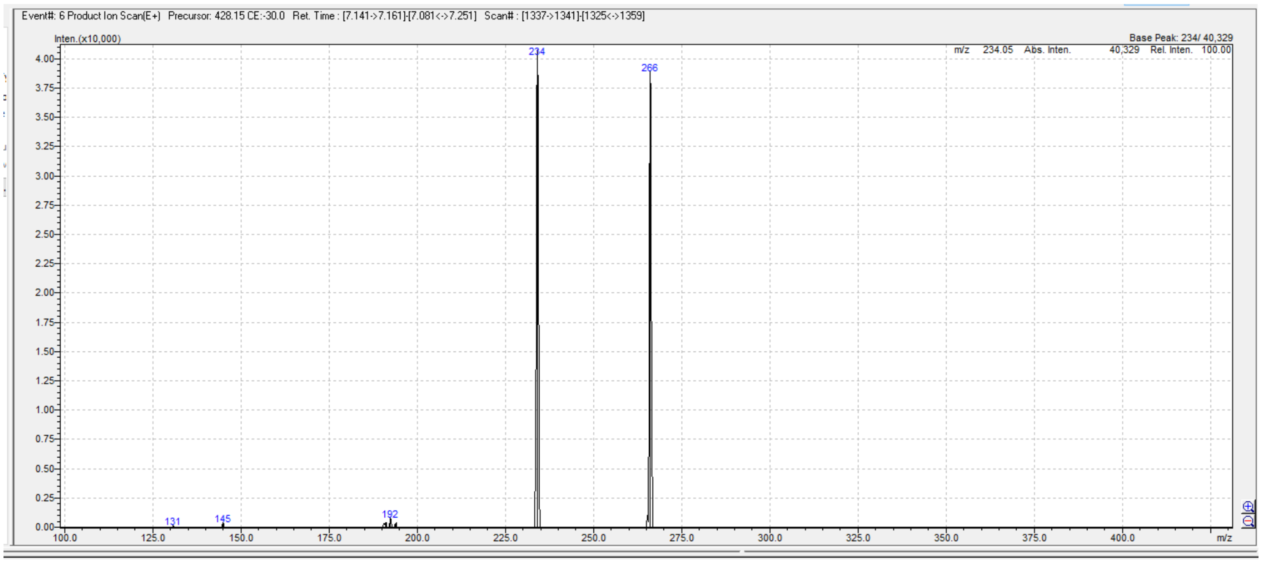

Mass Table

Mass Table

# of Peaks: 8

Raw Spectrum: [7.131->7.151],(scan:[1335->1339])

Background: [7.061<->7.281],(scan:[1321<->1365])

Base Peak: m/z 234.05 (Inten : 38,423)

m/z, (•) Abs. ( ) Rel. Intensity,

m/z	Intensity
130.80 ,	370
144.95 ,	418
148.50 ,	185
191.05 ,	333
192.40 ,	247
234.05 ,	38423
266.10 ,	36191
315.40 ,	123

C, FULL MASS SPECTRUM OF 460.14 CE -35 eV with fragmentation ion *m/z* 240, 198, 191 and 282

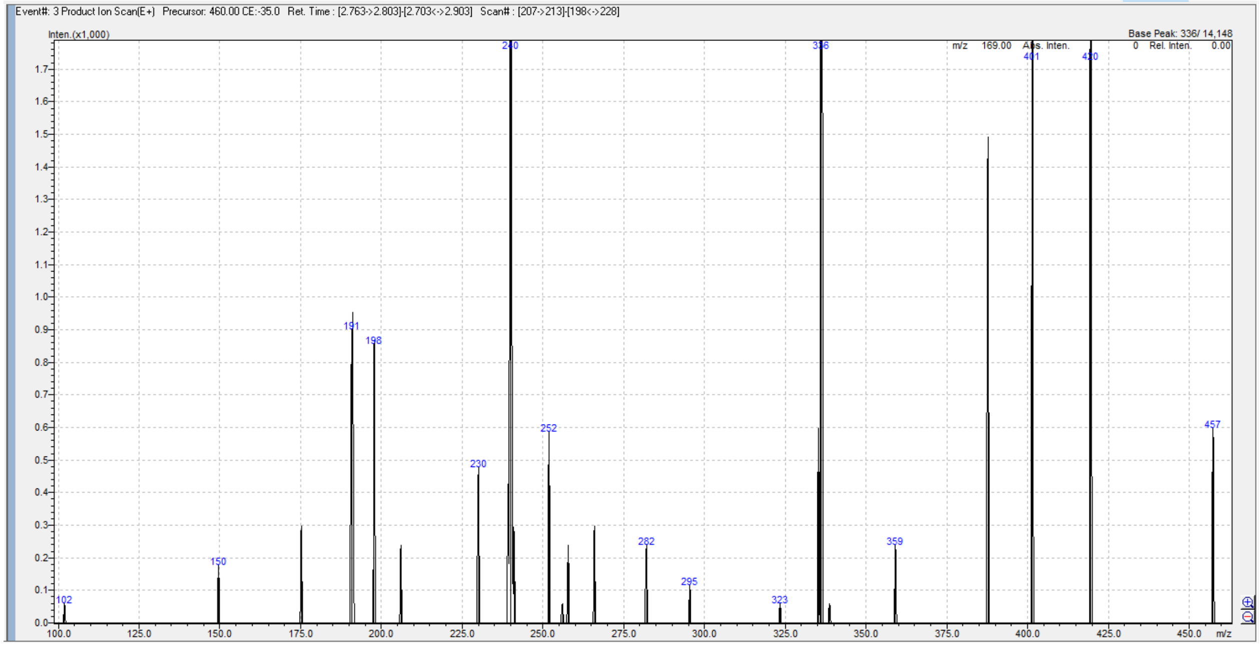

Mass Table

Mass Table

# of Peaks: 24

Raw Spectrum: [2.763->2.803],(scan:[207->213])

Background: [2.703<->2.903],(scan:[198<->228])

Base Peak: m/z 336.20 (Inten : 14,148)

m/z, (•) Abs. ( ) Rel. Intensity,

m/z	Intensity
101.90 ,	59
149.60 ,	178
175.20 ,	298
190.95 ,	900
197.75 ,	855
206.00 ,	238
230.00 ,	477
240.05 ,	2591
241.05 ,	283
251.90 ,	585

m/z	Intensity
256.00 ,	59
257.80 ,	238
265.90 ,	298
282.00 ,	238
295.45 ,	114
323.40 ,	59
335.30 ,	596
336.20 ,	14148
338.70 ,	59
359.10 ,	238

D, FULL MASS SPECTRUM OF 314.14 CE -25 eV with fragmentation ion *m/z* 238, 159

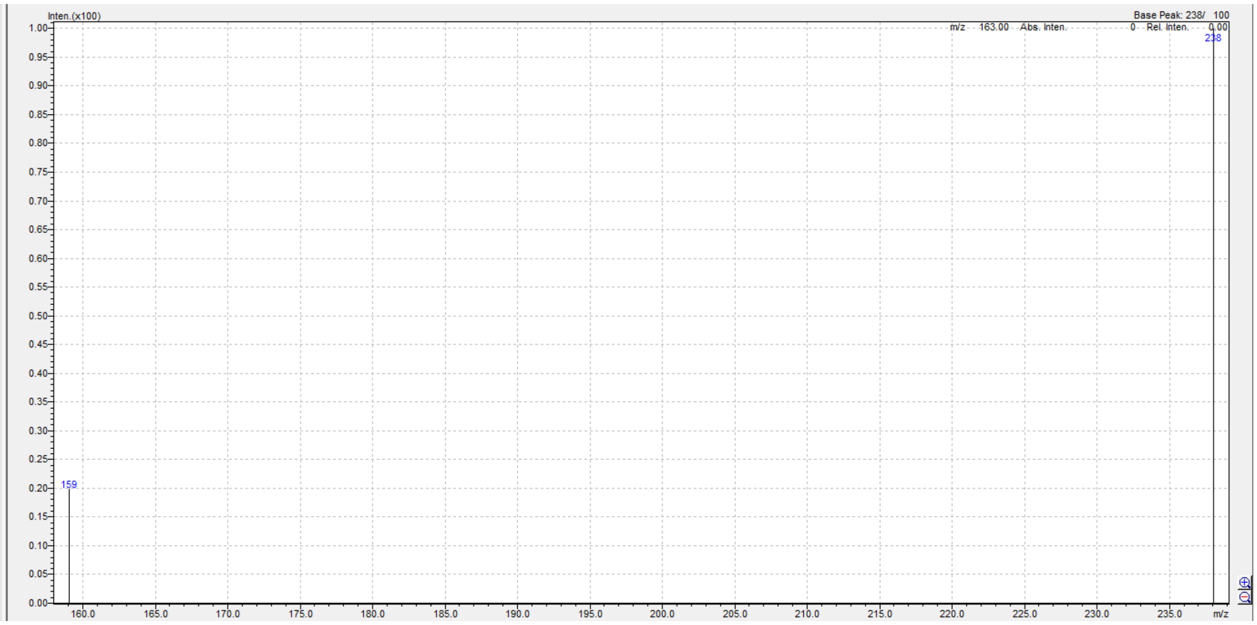

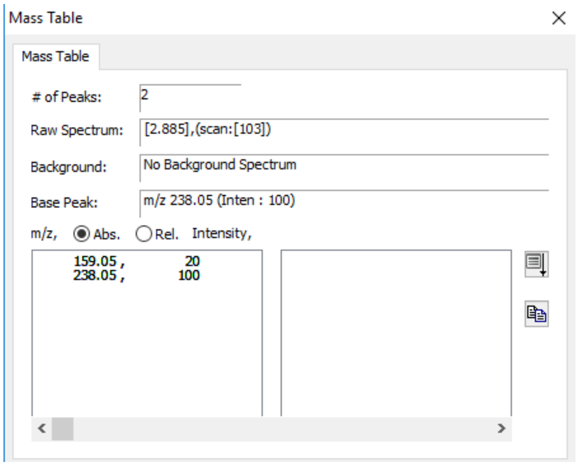

E, FULL MASS SPECTRUM OF 370.14 CE -30 eV with fragmentation ion *m/z* 208, 166

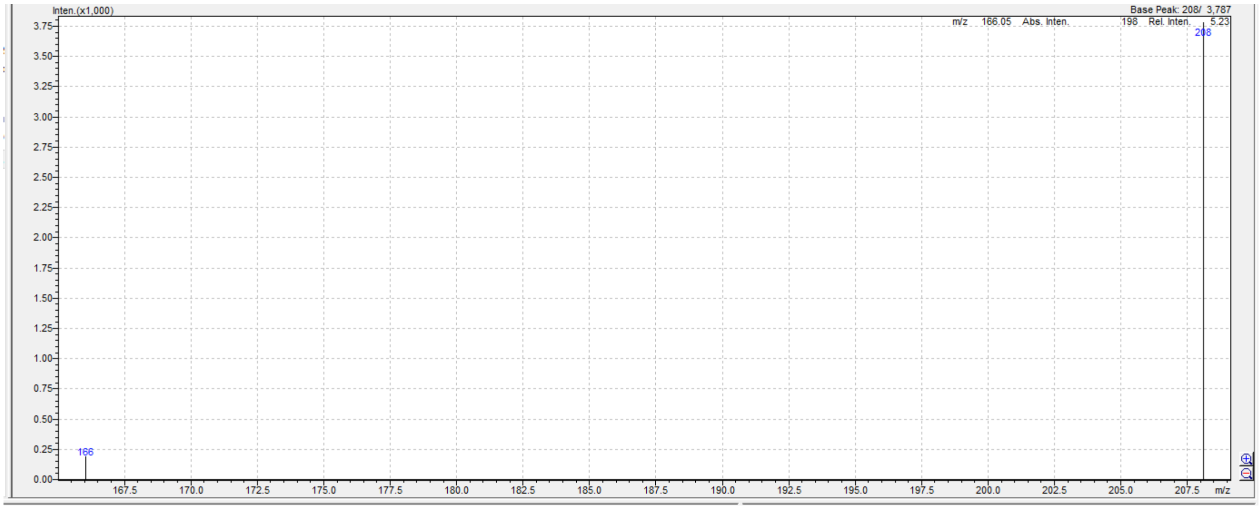

Mass Table

Mass Table

# of Peaks: 2

Raw Spectrum: [4.674->4.721],(scan:[318->324])

Background: [4.557<->4.861],(scan:[303<->342])

Base Peak: m/z 208.10 (Inten : 3,787)

m/z, Abs. Rel. Intensity,

m/z	Intensity
166.05 ,	198
208.10 ,	3787
